# Community-Acquired Pneumonia in Children: the Challenges of Microbiological Diagnosis

**DOI:** 10.1128/JCM.01318-17

**Published:** 2018-02-22

**Authors:** C. M. C. Rodrigues, H. Groves

**Affiliations:** a Department of Zoology, University of Oxford, Oxford, United Kingdom; b Department of Paediatric Infectious Diseases and Immunology, Great North Children's Hospital, Newcastle Upon Tyne, United Kingdom; c Centre for Experimental Medicine, Queens University, Belfast, United Kingdom; d Department of Paediatrics, Royal Belfast Hospital for Sick Children, Belfast, United Kingdom; Emory University

**Keywords:** bacterial, community-acquired pneumonia, diagnostics, molecular, PCR, viral

## Abstract

Community-acquired pneumonia (CAP) is the leading cause of mortality in children under 5 years of age globally. To improve the management of CAP, we must distinguish CAP from other common pediatric conditions and develop better diagnostic methods to detect the causative organism, so as to best direct appropriate resources in both industrialized and developing countries. Here, we review the diagnostic modalities available for identifying viruses and bacteria in the upper and lower respiratory tract of children, with a discussion of their utility and limitations in diagnosing CAP in children.

## INTRODUCTION

Community-acquired pneumonia (CAP) remains an important cause of morbidity and mortality in both industrialized and developing countries. Of all the children who died before their fifth birthday in 2013, pneumonia was the single most important disease, accounting for 14.9% (*n* = 935,000) of cases (1). However, despite being among the three most common infectious causes of death worldwide, pneumonia, diarrhea, and measles showed the greatest reductions between 2000 and 2013, suggesting that inroads are being made in preventing, recognizing, and treating these conditions. Improvements in access to health care, vaccination programs, living conditions, and nutrition are key to further reducing CAP mortality, and failure to do so is likely to disproportionately affect children in developing countries and directly influence their CAP incidence.

Traditionally, medical practitioners, having formulated a differential diagnosis from a constellation of clinical signs and symptoms, will utilize diagnostic tests to determine illness etiology. However, the diagnostic challenge of childhood CAP lies in the broad range of presenting features and the absence of an accepted gold standard diagnostic test. Furthermore, the diverse age range within pediatric practice adds to this challenge differences in immune development and vaccination status and reliance on caregivers for detailed patient histories. In addition, many diagnostic methods are initially validated in adult populations, which can make interpretation in the pediatric setting more difficult.

The definition of CAP varies between different sources; on a pathological level, pneumonia is considered infection of the lung parenchyma, i.e., lower respiratory tract (LRT) infection by microorganisms (2). CAP is defined clinically as “the presence of signs and symptoms of pneumonia in a previously healthy child due to an infection which has been acquired outside hospital” by both the British Thoracic Society (BTS) and the Infectious Diseases Society of America (IDSA), acknowledging that in resource-poor settings, chest X-rays (CXR) are not always available to aid diagnosis (3, 4).

## CLINICAL RECOGNITION OF CAP

Children can present with CAP at different stages of illness and with clinical features that are difficult to discriminate from other common pediatric diagnoses. Symptoms of CAP, including fever, cough, dyspnea, wheeze, chest or abdominal pain, lethargy, vomiting, and headache, can also be indicators of sepsis, congenital heart disease, profound anemia, malaria, or acute asthma (3), as can the typical examination findings of tachypnea, tachycardia, hypoxia, respiratory distress (grunting, nasal flaring, recession, and abdominal breathing), and crackles or wheeze on auscultation. The extent to which these signs are present with CAP is highly variable, which adds to the diagnostic complexity (Table 1).

**TABLE 1:** Clinical features of community-acquired pneumonia^*a*^

Degree of illness	Description of clinical features for:		
	Developing countries, all age groups	Industrialized countries	
		Infants	Older children
No CAP	No signs of pneumonia or severe pneumonia		
Mild or moderate		Temp <38.5°C	Temp <38.5°C
		RR <50/min	RR <50/min
		Mild recession	Mild dyspnea
		Taking full feeds	No vomiting
Severe	Fast breathing:	Temp >38.5°C	Temp >38.5°C
	≥50/min (2–11 mo)	RR >70/min	RR >50/min
		Moderate to severe recession	Moderate to severe recession
	≥40/min (1–5 yr)	Respiratory distress	Respiratory distress
		Tachycardia	Tachycardia
	Chest indrawing	Capillary refill time >2 s	Capillary refill time >2 s
		Intermittent apnea	Not taking full feeds
		Not taking full feeds	
Very severe	Cough or difficulty in breathing with:		
	Oxygen saturation <90% or central cyanosis		
	Severe respiratory distress (e.g., grunting, very severe chest indrawing)		
	Signs of pneumonia with a general danger sign (inability to breastfeed or drink, lethargy or reduced level of consciousness, convulsions)		

a Clinical features of community-acquired pneumonia (CAP) as described by the World Health Organization (WHO) for diagnosis of CAP in developing countries (7) and by British Thoracic Society Guidelines applicable for infants and older children in industrialized countries (3). RR, respiratory rate.

Historically, World Health Organization (WHO) guidance on recognition of pneumonia relied on tachypnea as an indicator of CAP requiring treatment with oral antibiotics, prioritizing sensitivity over specificity to avoid missing cases of disease in settings where late diagnosis could result in increased mortality. Such an approach may lead to overdiagnosis, as demonstrated in an observational study in four Indian hospitals. Follow-up of 516 children diagnosed with WHO-defined pneumonia at presentation who were reassessed by pediatricians 4 days later only found 35.9% to have pneumonia, and the remainder were recategorized with wheeze (42.8%), mixed disease (18.6%), and nonrespiratory illness (2.7%) (5). Accordingly, this approach does not discriminate between pulmonary pathologies and may lead to overuse of antibiotics. Indeed, research into use of the WHO guidelines in low-income countries has identified overdiagnosis of pneumonia in cases of wheezing, with consequent underdiagnosis of asthma, leading to significant respiratory morbidity and, perhaps, even mortality (6).

However, the benefit of the updated WHO guidance for CAP lies in the use of simple clinical signs to direct optimal antibiotic therapy. For instance, children aged 2 to 59 months with cough and/or difficulty breathing can be treated with oral amoxicillin in the absence of red flag signs, which include inability to drink, persistent vomiting, seizures, lethargy, reduced consciousness, stridor, or severe malnutrition (7). Industrialized countries typically have greater access to CXR as a diagnostic adjunct in children admitted to hospital, with consolidation, infiltrates, and air bronchograms visible in a lobar or diffuse pattern. The value of chest radiography is clear in excluding complications like pleural effusion, necrotizing pneumonia, or other diagnoses, including cardiac failure with pulmonary edema. However, it is important to note that clinical signs and chest radiography often have poor agreement in ambulatory patients, and thus, the BTS guidelines do not recommend routine CXR in suspected childhood CAP patients who are managed in the community (3). Nevertheless, attempts have been made to correlate clinical findings with radiological evidence of pneumonia for the development of improved clinical tools to use in resource-poor settings. United Kingdom and U.S. studies show that tachypnea has the greatest correlation and that additional symptoms, such as dyspnea/hypoxia or fever/hypoxia, may increase sensitivity (8, 9). A meta-analysis of 18 studies from low-, middle-, and high-income countries identified the best prediction of radiological pneumonia as being achieved using a combination of the following clinical signs: tachypnea of >50/min at any age, grunting, chest in-drawing, and nasal flaring (10). We have already highlighted the challenge in defining a reference standard for clinical CAP diagnosis, and accordingly, studies in this meta-analysis display considerable heterogeneity, thereby limiting the interpretation of findings.

## ESTABLISHING CAP ETIOLOGY

CAP can be caused by viruses, bacteria, or both. These causative agents are indistinguishable on the basis of clinical features alone; the diagnostic difficulty is primarily due to the inability to isolate the causative organism from the lower respiratory tract, as few young children have productive sputum or positive blood sample cultures (3). Older children and adults can produce sputum for examination under microscopy and culture. This is much more difficult in younger children, who typically do not expectorate. Table 2 outlines the range of viral and bacterial pathogens isolated from cases of childhood CAP in six studies worldwide; descriptions of the studies are given in Table 3 (11–16). Interestingly, studies from The Gambia, India, and the United Kingdom appeared to have higher proportions of Streptococcus pneumoniae isolated, which suggests a potentially region-specific etiology for childhood CAP.

**TABLE 2:** Distribution of pathogens identified from children with CAP within different global regions^*a*^

Pathogen	% of patients positive for pathogen in:					
	United Kingdom	United States	Kenya	The Gambia	Nigeria	India
Viruses						
RSV	21.2	28.0	34	4.0	30.4	24.1
Rhinovirus	8.5	27.0	NT	—	—	10.5
hMPV	0.7	13.0	3.0	—	—	2.8
Influenza virus	7.4 (A, B)	7.0 (A, B)	5.8 (only A)	2.0 (only C)	17.3 (only A)	3.5 (A, B, C)
Bocavirus	3.3	—	—	4.0	—	—
Adenovirus	6.9	11.0	3.8	4.0	—	3.7
Parainfluenza virus	4.3 (types 1–4)	7.0	3.8 (type 3)	—	19.5 (type 3)	7.5 (types 1–4)
Bacteria						
S. pneumoniae	17.4	4.0	NT	91.0	5.1	5.7
H. influenzae	2.3	—	NT	23.0	—	0.8
Group A Streptococcus	10.5	1.0	NT	—	—	—
S. aureus	2.3	1.0	NT	6.0	37.3	0.8
M. pneumoniae	9.9	8.0	NT	—	—	4.3 (serology)
Moraxella catarrhalis	2.3	—	NT	—	—	—
Klebsiella pneumoniae	0.8	—	NT	—	15.3	0.2

a Pathogens from children with CAP within different global regions were identified using a variety of samples obtained from the patients as part of clinical and research studies and tested using both traditional culture and molecular tests; the studies are described in Table 3. NT, not tested; —, results for these organisms were not available in the respective studies; RSV, respiratory syncytial virus; hMPV, human metapneumovirus.

**TABLE 3:** Studies of pathogen detection in children with CAP within different global regions^*a*^

Region	Specimen types and laboratory tests used^*b*^	Study size	Age of children	Reference
United Kingdom	Blood culture, blood pneumococcal real-time PCR, NP PCR, pleural fluid culture/pneumococcal antigen testing/PCR, ETT aspirate/BAL fluid for culture/PCR	160	0–16 yr	11
United States	Blood cultures, whole-blood PCR, NP/OP PCR, pleural fluid culture/PCR, BAL fluid or ETT aspirate culture	2,222	<18 yr	12
Kenya	Blood culture and nasal wash fluid for real-time PCR and DNA sequencing	759	1 day–12 yr	13
The Gambia	Lung and pleural aspirate culture for nonmolecular serotyping, singleplex and multiplex PCR, 16S rRNA PCR, MLST, molecular serotyping	53	2–59 mo	14
Nigeria	Blood culture, IFA, serology	205 blood cultures, 122 viral tests	<5 yr	15
India	Blood culture, BAL fluid culture/PCR, NPA culture/PCR/multiplex PCR, serology	2,285 blood culture, 2,323 NPA, 428 NPA multiplex PCRs	1 mo–12 yr	16

a Data from the studies are given in Table 2.

b The specimen types and laboratory tests used for analysis of pathogen detection are listed. NP, nasopharyngeal; OP, oropharyngeal; IFA, immunofluorescence analysis; BAL, bronchoalveolar lavage; ETT, endotracheal tube; MLST, multilocus sequence typing.

## VIRAL DIAGNOSTIC TECHNIQUES

Clinical virology diagnosis has been revolutionized over the past 2 decades with the introduction of nucleic acid-based detection. The majority of respiratory tract infections in children are viral in origin, and both the BTS and IDSA guidelines for management of childhood CAP recommend viral testing of nasopharyngeal secretions and/or nasal swabs by PCR or immunofluorescence (3, 4). PCR has been demonstrated to have greater sensitivity than virus isolation in cell culture, shell vial culture, and immunofluorescence testing and is now the mainstay of respiratory virus detection in industrialized countries (17). While rapid antigen detection testing (RADT) for respiratory syncytial virus (RSV) and influenza virus is still in conventional use due to its low cost and fast results, this technique has relatively poor sensitivity in comparison to that of nucleic acid-based detection methods (18).

Thus, multiplex PCR, enabling the detection of numerous pathogens simultaneously without additional time or sampling, is used extensively (17, 19). The results of multiplex PCR assays are rapid and typically available within 1 to 6 h, and as the availability of this technology grows, competition is decreasing prices, making the technology more affordable, which is essential for implementation in the developing world setting (19). Indeed, some laboratories are developing custom kits with performance comparable to that of commercial ones at a much-reduced cost (20). It is worth noting, however, that despite this, multiplex PCR is an expensive technology, and while publications often cite the use of over 20 targets, the selection of multiplex kits is based on a range of factors, including local expertise, funding structures, and the panel of pathogens detected, which has the disadvantage of leading to variations in practice between hospital centers (21).

Following the introduction of multiplex PCR technology in routine diagnosis of childhood CAP, the presence of multiple viral agents is more commonly seen, with rates of 30 to 40% and up to four different viruses present in individual children (17). The significance of this remains unclear. For particular viral pathogens, such as RSV infection, it is understood that coinfection with other respiratory tract viruses can worsen disease severity. However, there is conflicting evidence regarding the impact of other viral coinfections on the severity of respiratory tract infections. Additionally, it is worth noting that among healthy controls tested, PCR can also be positive for one or more viruses. These findings may be explained by the high number of infections occurring in children in quick succession, with overlapping viral shedding. However, it also highlights a potential pitfall of PCR for diagnosing etiological pathogens, as the challenge remains to establish whether a detected virus is causing or associated with CAP or indeed simply represents carriage/colonization (19). This is exemplified by the recently identified human bocavirus (hBoV), which has been detected in children with lower respiratory tract disease (reported rates range from 1.5% to 13%). With up to an 83% coinfection rate, it is uncertain whether hBoV is indeed an etiologic agent, an exacerbating factor, or an incidentally detected bystander (22). Notably, for a number of viruses, detection in asymptomatic children is very infrequent (influenza, 0%; RSV, 1.9%; and human metapneumovirus [hMPV], 1.5%), and therefore, it is likely that the presence of one of these viruses in a symptomatic individual is highly suggestive of an etiological role (23). The use of copy number/cycle threshold (*C_T_*) in quantitative real-time PCR (qRT-PCR) as a semiquantitative estimate of viral load value has been explored to assess the clinical significance of a detected virus. With rhinovirus infections in childhood, a higher viral load (lower *C_T_* value) in nasal swab samples has been associated with increased likelihood of LRT infection (24). However, there are several factors that can influence *C_T_* values, including variations in the period of viral shedding and differences in sampling and laboratory techniques, and thus, the full role of this technique in daily clinical practice is unclear at the time of writing.

Determining viral etiology is still more problematic in countries without routine molecular diagnostic facilities. Immunofluorescence, serology, and viral culture have been used previously; however, these may underestimate the burden of viral CAP. A Kenyan study used PCR methods on nasal washing samples and identified viruses in 425 of 759 children with clinically very severe/severe pneumonia (Tables 2 and 3) (13). Studies in children have demonstrated high specificity and negative predictive values for the detection of parainfluenza and adenovirus in nasopharyngeal aspirate samples, but discordance remains between bronchoalveolar lavage and nasopharyngeal aspirate samples in the detection of bacterial infections (25). However, the paired sample numbers included in these studies are relatively small, making the true agreement unclear, and further work is needed on the implications for clinical management.

In view of the limited availability of antiviral therapies for respiratory diseases, specific viral identification may be considered unnecessary, as for most cases, supportive therapy alone is sufficient. However, the clinical benefits of rapid and specific microbial identification of CAP include optimizing antibiotic use and reduction in nosocomial transmission through effective patient cohorting (26). A Cochrane review of rapid viral PCR diagnosis did not demonstrate reduced antibiotic use in an emergency department setting; however, a more recent large, single-center study in New York, demonstrated that the implementation of multiplex PCR testing resulted in less antibiotic usage and reduced chest radiography (27, 28). The findings at this referral pediatric hospital may not be generalizable to all pediatric care settings, but they highlight a promising benefit of novel viral diagnostic testing, and certainly, the clinical impact of multiplex PCR requires further evaluation. Furthermore, as new specific antiviral therapies, such as novel RSV therapies, undergo clinical trials, the accurate diagnosis of viral etiology will become increasingly important for children who become extremely unwell or who are immunocompromised (29).

## BACTERIAL DIAGNOSTICS TECHNIQUES

It is well-accepted that bacterial infection commonly follows viral infection; although the pathogenesis is not fully elucidated, it is thought to relate to inflammation arising secondary to viral infection (4). The most common pathogens include Streptococcus pneumoniae, Haemophilus influenzae (including nontypeable strains), and Staphylococcus aureus. Atypical causes include Mycoplasma pneumoniae, Chlamydia pneumoniae, and Legionella pneumophila. For accurate pathogen identification, the principle of obtaining a sample directly from the lung not contaminated by host flora would be optimal. Lung aspiration provides such a sample; it is invasive and rarely performed but has historically contributed significantly to the understanding of bacterial causes of CAP. Using molecular diagnostics as well as bacterial culture can increase the diagnostic benefits of lung aspiration. In a study of 55 Gambian children with clinically or radiologically confirmed CAP, lung aspiration samples were tested using culture and molecular techniques (single/multiplex PCR and multilocus sequence typing). By additionally applying molecular methodology to cultures of 53 lung aspirate and pleural fluid samples, the identification of an organism increased, with samples yielding 91% S. pneumoniae, 23% H. influenzae, and 6% S. aureus. Interestingly, viral identification alone in these LRT samples was extremely low, at 2%, compared with the rates of identification in the previous studies sampling the nasopharynx. Bacterial and viral codetection was noted in 19% of samples, while bacterial-bacterial codetection was more likely, at 40%, with S. pneumoniae and H. influenzae at 21% (14). Interpretation of the presence of these potentially pathogenic organisms in the lungs of children with radiological CAP remains challenging, as pathogen detection alone cannot confirm causation. In this regard, we may achieve greater insight and interpretation of studies of lung aspirate samples with the increasing understanding of the lung microbiome.

Routine microbiological investigations for bacterial causes of CAP include blood culture, sputum culture, serology for atypical bacteria (Mycoplasma spp. and Chlamydia spp.), and pneumococcal antigen detection/PCR, as well as culture of pleural fluid where samples are available. The role of blood culture in CAP diagnostics is limited. A recent meta-analysis found that only 9.89% of blood cultures taken are positive in hospitalized children with severe CAP, with substantial false-positive rates (30). These results are perhaps unsurprising, given that cultures may be taken with concomitant antibiotic use and infection is generally localized to lung parenchyma. In fact, a study undertaken by Andrews et al. noted that universal blood sample culturing would require 118 blood samples to be taken in order to identify a single bacteremia that would result in a meaningful antibiotic change (31). This supports both BTS and IDSA CAP guidelines advocating the use of blood sample cultures only in patients with severe CAP admitted to intensive care or with complications, due to its wide availability, the difficulty of confirming clinical and radiological diagnoses, and the potential for organism identification and antibiotic sensitivity information in these high-risk children (3, 4).

As discussed previously, sputum culture is challenging to achieve in young children but has been shown to be of benefit in children hospitalized with CAP. The use of induced sputum production via the administration of hypertonic saline using a nebulizer, followed by chest wall percussion, is generally well tolerated, although coughing and wheezing can occur. However, this procedure can result in contamination with upper respiratory tract (URT)-colonizing organisms, leading to false interpretations of pathogenesis. One way to avoid such contamination is the use of bronchoalveolar lavage and culture, but this procedure is very invasive and is therefore limited to specialized units and intensive care settings. In addition to microbiological culture, the developing role of matrix-assisted laser desorption ionization–time of flight mass spectrometry (MALDI-TOF MS) in clinical laboratories is allowing rapid and accurate identification of organisms that may previously have been interpreted as pathogenic. This still requires the growth of a bacterial colony as an input sample but may help identify commensal bacteria with more certainty than traditional biochemical testing, preventing inappropriate antibiotic use. It must be noted, however, that the results are limited by the reference databases, which require regular updating (32).

Serological testing in pneumonia, performed 14 days apart, is still considered the gold standard for Mycoplasma pneumoniae detection, but this is complex clinically, and in practice, treatment is often commenced empirically based on clinical suspicion (33). Similarly, pneumococcal serology is also considered too complex for routine clinical use, and obtaining convalescent-phase samples does not alter the management of acute CAP. Detection of pneumococcal antigen in urine has low specificity in young children (3).

In view of these challenges in identifying a causative bacterial agent, a pragmatic approach of therapy with broad-spectrum antibiotics is typically employed. While advantageous clinically, in this era of emerging antibiotic resistance, the identification of specific bacteria may prove beneficial. As with viral diagnostics, the use of PCR is a major development in the detection of respiratory bacterial pathogens. In fact, multiplex PCRs for throat and nasal swabs that include a panel of viruses as well as bacterial pathogens (e.g., Mycoplasma pneumoniae or Bordetella pertussis) are now being used to increase etiological yield in CAP (34). The employment of this technology has revealed high rates of bacterial and viral coinfection, the significance of which is a source of ongoing investigation, in the pediatric setting in particular. While molecular testing has greatly improved sensitivity in the detection of bacterial pathogens in CAP, its role in discriminating between infection and colonization is less clear. For example, in a recent study of Mycoplasma pneumoniae, 21.2% of asymptomatic children had positive mycoplasma PCR testing (35). Although a small, single-center study, this result highlights the diagnostic challenge this new technology presents. Further studies on the significance of these detected pathogens and correlation with clinical findings are needed to help differentiate carriage from infection.

Furthermore, new molecular techniques, such as multilocus sequence typing of bacterial isolates, have an emerging role in epidemiological tracking of hospital and community outbreaks of bacterial CAP, as well as in the characterization of antibiotic resistance mechanisms and insights into the carriage and transmission of organisms. At the time of writing, this work was largely restricted to the research setting, but in future, it will provide large-scale surveillance data regarding the organisms that cause bacterial CAP, in particular changes in S. pneumoniae carriage and disease in the context of vaccination (36).

## FUTURE INSIGHTS FOR DIAGNOSTICS

As detailed above, the development of nucleic acid-based detection methods has dramatically altered the microbiological diagnosis of CAP. Future research is required to understand viral and bacterial colonization of the respiratory tract and the relevance of the detection of multiple viral agents in CAP pathogenesis, with consideration given to consecutive versus simultaneous detection of multiple pathogens. Across both the developed and the developing world, greater vaccine coverage against H. influenzae type b and pneumococcus is contributing to alterations in the epidemiology of bacterial CAP, and viruses are increasingly recognized as a substantial cause of CAP. Accordingly, point-of-care (POC) tests to accurately differentiate between viral and bacterial pneumonia are urgently needed. Several tests that employ either real-time PCR or isothermal amplification technology are being developed for POC testing of childhood infectious diseases (Table 4). Integration of the steps required for POC real-time PCR has been developed in the Cepheid GeneXpert and the Roche IQuum Liat analyzers. Indeed, the GeneXpert MTB/RIF test for detection of M. tuberculosis complex and rifampin resistance has been endorsed by the WHO for POC testing of tuberculosis (TB) resistance. However, these instruments are expensive to purchase and require complex sample preparation to mitigate the risk of PCR inhibition, which may limit the availability, utility, and therefore, implementation of the tests in resource-poor settings worldwide or in primary care (37).

**TABLE 4:** Summary of current active clinical trials on the use of molecular testing for childhood CAP^*a*^

Study identifier	Test type^*b*^	Study summary/measures^*b*^	Age group	End date
NCT02957136	POC diagnostic test	RCT to assess effect of near-POC testing on antibiotic and anti-influenza medication use in ED patients (FilmArray respiratory panel; Biofibres Diagnostics, LLC)	1–101 yr	Aug 2018
NCT02018198	POC diagnostic test	Single group assignment diagnosis study to investigate FebriDx POC diagnostic test vs standard assessment in febrile URTI	>2 yr	May 2017
NCT02668237	Multiplex PCR/urinary test	Use of multiplex PCR and antigenic urinary test diagnostic strategy vs standard in ED	3 mo–18 yr	Jun 2016
NCT03075111	POC diagnostic test	Retrospective external validation of novel IVD assay for differentiation of bacterial vs viral etiology of patients with acute febrile disease	3 mo–18 yr	Dec 2018
NCT03029299	POC diagnostic test	Randomized crossover intervention study measuring time duration from initial visit to receipt of appropriate therapy following implementation of the FilmArray RP EZ POC test	0–100 yr	Jun 2017
NCT02929680	Respiratory panel test	Prospective clinical evaluation of the FilmArray LRTI panel vs culture (BioFire Diagnostics)	Child, adult, senior	Dec 2017
NCT03052088	POC diagnostic test	Prospective clinical validation of sensitivity/specificity of novel (CE-IVD marked) diagnostic assay (ImmunoXpert) in differentiating bacterial vs viral etiologies in pediatric patients with suspicion of respiratory tract infection	>3 mo	Jul 2019
NCT00342589	Oral wash PCR testing	Study to examine effectiveness of PCR on samples obtained using a simple oral wash for diagnosis of pneumocystis infection	3–99 yr	Jul 2018
NCT02880384	PCR panel	Study to compare no. of CAP pathogens detected using current diagnostic bundle vs no. detected using FilmArray LRTI version 2.0 IUO PCR panel (BioFire Diagnostics)	Child, adult, senior	Dec 2018
NCT02851771	POC diagnostic test	Interventional single group study using POC testing to expand the etiological diagnosis strategy of pneumonia	Child, adult, senior	Oct 2019
ISRCTN66872125	Multiple test modalities	Prospective study on etiology, diagnostics, clinical management, impact, and outcomes of SLS and ARI across Europe	<6 yr	Dec 2018

a Data were obtained from searches of UK Clinical Trials Gateway, EU Clinical Trials Register, ISRCTN registry, International Clinical Trials Registry Platform (ICTRP) Search Portal, and ClinicalTrials.gov online databases.

b POC, point of care; RCT, randomized controlled trial; ED, emergency department; URTI, upper respiratory tract infection; IVD, *in vitro* diagnostic; LRTI, lower respiratory tract infection; RP, respiratory panel; SLS, sepsis-like syndrome; ARI, acute respiratory tract infection.

Therefore, the development of novel amplification technologies is vital to address these limitations. One such recent development is the loop-mediated isothermal amplification (LAMP) method, where samples are amplified without the need for thermal cycling (38). This provides many advantages over PCR, including a simplified procedure, reduced time to detection, and more compact, less expensive detection equipment. Several LAMP assays have recently been validated with performance comparable to that of PCR, including LAMP assays for the detection of S. pneumoniae and group B Streptococcus, as well as a pertussis assay, which was noted to be 2.5 times faster than real-time PCR, with sensitivity of 96.55% and specificity of 99.46% (39). This technology, therefore, may prove invaluable in POC microbiology in developing countries; however, further optimization is required to enhance sensitivity in respiratory virus detection and in the detection of multiple pathogens.

To assess current research directions for molecular testing in childhood CAP, we performed a comprehensive search of all active clinical trials registered in the United Kingdom, European, WHO, and U.S. clinical trial databases. This strategy identified 11 current trials involving molecular testing for childhood CAP, which are summarized in Table 4. Due for completion by the end of 2019, these studies include POC testing and clinical applicability trials for directing patient therapy/management. The results of these and future trials may answer some of the questions surrounding the clinical application of molecular testing in microbial diagnosis and help inform clinical practices regarding their role in the diagnosis and management of childhood CAP. With the current significant limitations of diagnostics in CAP, the advent of new technologies and the prospect of rapid POC testing are very exciting. For the clinician, the ability to rapidly diagnose CAP and to distinguish at diagnosis the specific etiological agent, whether bacterial, viral, or both, would prove invaluable in directing the appropriate use of antibiotics and is likely to transform the way we deliver care to these children in future.
